# Ventilation before Umbilical Cord Clamping Improves the Physiological Transition at Birth

**DOI:** 10.3389/fped.2014.00113

**Published:** 2014-10-20

**Authors:** Sasmira Bhatt, Graeme R. Polglase, Euan M. Wallace, Arjan B. te Pas, Stuart B. Hooper

**Affiliations:** ^1^The Ritchie Centre, MIMR-PHI Institute of Medical Research, Monash University, Melbourne, VIC, Australia; ^2^Department of Obstetrics and Gynaecology, Monash University, Melbourne, VIC, Australia; ^3^Department of Pediatrics, Leiden University Medical Centre, Leiden, Netherlands

**Keywords:** neonatal, preterm birth, transition, umbilical cord clamping, delayed cord clamping

## Abstract

The transition from a fetus to a neonate at birth represents a critical phase in our life. Most infants make this transition without complications, but preterm infants usually require some form of assistance due to immature cardiopulmonary systems that predispose them to lifelong sequelae. As the incidence of preterm birth is increasing, there is now an urgent need for the development of management strategies that facilitate this transition, which will likely include improved strategies for the management of the maternal third stage of labor. For instance, recent studies on the physiological transition at birth have led to the discovery that establishing ventilation in the infant before the umbilical cord is clamped greatly stabilizes the cardiovascular transition at birth. While most benefits of delayed clamping previously have been attributed to an increase in placenta to infant blood transfusion, clearly there are other significant benefits for the infant, which are not well understood. Nevertheless, if ventilation can be established before cord clamping in a preterm infant, the large adverse changes in cardiac function that normally accompanies umbilical cord clamping can be avoided. As preterm infants have an immature cerebral vascular bed, large swings in cardiovascular function places them at high risk of cerebral vascular rupture and the associated increased risk of mortality and morbidity. In view of the impact that cord clamping has on the cardiovascular transition at birth, it is also time to re-examine some of the strategies used in the management of the third stage of labor. These include the appropriate timing of uterotonic administration in relation to delivery of the infant and placenta. As there is a lack of evidence on the effects these individual practices have on the infant, there is a necessity to improve our understanding of their impact in order to develop strategies that facilitate the transition to newborn life.

## Introduction

One of the first major interventions that an infant experiences following birth is umbilical cord clamping (UCC) and its separation from the placenta. This signifies a landmark period during which the newborn transforms into an independent entity. Much interest has recently focused on the appropriate timing of UCC, particularly on the risks and benefits of delaying cord clamping for a set period of time after birth. Debate on appropriate timing of UCC following birth has been ongoing for several decades (even centuries), yet the ideal time still remains unknown. As a result, although the potential benefits of delayed umbilical cord clamping (DCC) have been documented, ‘early’ or ‘immediate’ umbilical cord clamping (ICC) is the most widely used procedure and is part of the active management of third stage of labor; i.e., the period extending from complete delivery of the infant to complete delivery of the placenta (1, 2). Although the reasons for this are unclear, a lack of understanding and awareness of the issues associated with UCC are thought to be a major underlying factor (3, 4).

The benefits and risks associated with DCC have been primarily attributed to an increase in neonatal blood volume, secondary to placento-fetal transfusion. But this does not readily explain all observations commonly associated with DCC, for example the lower risks of cerebral hemorrhage and necrotizing enterocolitis (5). These benefits appear more in line with improvements in cardiac function, which may or may not be associated with an increase in blood volume. Recent evidence suggests that DCC until after ventilation onset maintains ventricular preload and stabilizes cardiovascular function during the transition, which provide an alternative explanation for the lower risk of cerebral hemorrhage (5, 6). That study highlighted the need to consider DCC with respect to the physiological changes that occur within the infant during its transition after birth, although this area has received relatively little attention. This article is focused on the science underpinning the physiological changes at birth and how the timing of UCC may influence these changes. Recently, an excellent review on DCC has been published (5), which details much of the clinical data published on the risks and benefits of DCC and so will not be repeated here.

## History

Delayed umbilical cord clamping is not a modern concept, as some primitive cultures reportedly wait until well after delivery of the placenta before cutting the umbilical cord. Indeed, Erasmus Darwin in 1801, suggested that, *“*Another thing very injurious to the child, is the tying and cutting of the navel string too soon; which should always be left till the child has not only repeatedly breathed but till all pulsation in the cord ceases. As otherwise the child is much weaker than it ought to be.” (7). Similarly, numerous other scholars, dating from the 19th century have provided similar observations and opinions about the timing of UCC at birth (8). However, these opinions were based on observational studies and not on scientific evidence. Nevertheless, it seemed natural to these scholars to leave the cord intact until after the child had taken its first breath. This raises the question as to why ICC currently predominates. While some have attributed this to maintaining “custom,” other reasons include reducing the risk of post-partum hemorrhage (PPH), reducing neonatal blood loss before physiological closure of the cord, easier identification of placental detachment, minimizing the risks of rhesus iso-immunization, and time constraints amidst a busy and chaotic delivery suite environment (9, 10).

## Current Guidelines on the Timing of Umbilical Cord Clamping

Guidelines on the timing of UCC vary considerably world wide, although ICC is the most commonly used practice. However, as DCC is not associated with an increased incidence of adverse maternal effects, including PPH, and may confer potential benefits for the infant, recent international guidelines now recommend DCC (11). In 2010, the International Liaison Committee on Resuscitation recommended that UCC be delayed for at least 1 min in healthy term infants not requiring intervention (11, 12), but is contraindicated for neonatal asphyxia (12). Instead, it is recommended that the asphyxic infant is separated from the placenta and transferred to a resuscitation table for urgent resuscitation, although this recommendation is not based on scientific or clinical evidence (12). Indeed, it could be argued that these infants would receive the greatest benefit from DCC, especially if delayed until respiration is initiated.

## Birth Transition; the Physiology

Birth represents a major physiological challenge for the fetus as it transitions from a liquid-filled *in utero* environment to a gaseous *ex utero* environment (13, 14). Although most babies undergo this transition without difficulty, some infants, mainly preterm infants (~10% of all births), require substantial assistance most commonly in the form of respiratory support (13). This is because their lungs are immature and the circulatory changes that facilitate adaptation to extra-uterine life can be delayed. As a result, these infants are at increased risk of developing BPD, cardiovascular sequelae including persistent pulmonary hypertension of the newborn, and/or intra-ventricular hemorrhage (IVH); all of which are associated with poor neurodevelopmental outcomes (15, 16).

During fetal life, the lungs are liquid-filled and gas exchange occurs across the placenta (17, 18). Thus, following separation of the infant from the placenta after birth, the lungs must take over the role of gas exchange. To do this, the airways must be cleared of liquid to allow the entry of air into the distal gas-exchange regions and blood flow through the lungs must markedly increase (14, 19). Teleologically, it is logical that these two events are linked. Indeed, it is now well established that air entry into the lungs triggers a decrease in pulmonary vascular resistance (PVR) and increase in pulmonary blood flow (PBF). While the entry of gas into the lung is the primary driver for the increase in PBF, the exact mechanisms that sense and mediate this event remain unclear (20). Increased oxygenation, mediated by NO release (20), is one component of the response, but mechanisms that are independent of oxygen are also involved (21, 22).

### Lung aeration

Until recently, the mechanisms thought to regulate airway liquid clearance at birth centered on Na^+^ reabsorption, but it is now recognized that it can occur via a number of mechanisms, with the relative contribution of each depending upon the timing and mode of delivery (23–25).

#### Changes in transpulmonary pressure

Changes in fetal posture, particularly increased fetal trunk flexion caused by amniotic fluid loss, greatly increase transpulmonary pressures by increasing abdominal pressure and elevating the diaphragm (26). Thus, following labor onset and membrane rupture, the forces imposed on the fetus by uterine contractions likely impose major changes in fetal posture that result in large increases in transpulmonary pressure (13, 19). As the fetal respiratory system is very compliant, large increases in transpulmonary pressure will force liquid from the lungs via the trachea (17), which likely explains the large “gushes” of liquid from the nose and mouth that have been commonly reported following delivery of the head (14).

#### Na^+^ reabsorption

With the onset of labor, increased fetal adrenaline (and arginine vasopressin) levels caused by the stress of labor are thought to activate epithelial Na channels, which increases Na^+^ and Na^+^-linked Cl^−^ flux across the pulmonary epithelium (27). This reverses the osmotic gradient and the direction of liquid movement across the epithelium (27). However, this mechanism only develops late in gestation, is not active in preterm infants, and reabsorption rates induced by pharmacological doses of adrenaline are orders of magnitude slower than that required to aerate the lung (19, 23, 24).

#### Transpulmonary pressures generated by inspiration

After birth, airway liquid clearance primarily results from transepithelial pressure gradients generated by inspiration (19, 23, 24). Phase contrast X-ray imaging studies have shown that lung aeration and airway liquid clearance only occurs during inspiration, with little or no clearance between breaths (23–25). Based on these findings, it was concluded that airway liquid clearance primarily results from transepithelial pressure gradients generated by inspiration; i.e., expansion of the thoracic cavity decreases interstitial tissue pressures below airway pressures. As a result, liquid moves from the airways into the tissue, which increases resting interstitial tissue pressures (28) and chest wall expansion (23). This mechanism of airway liquid clearance continues after birth and assists, along with other mechanisms (e.g., expiratory braking) in maintaining end-expiratory lung volumes (25, 29).

### Cardiovascular transition

**Figure 1:**
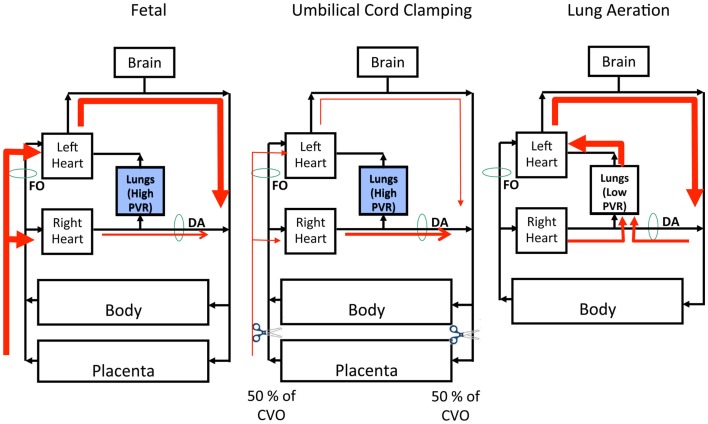
**Left Panel: The fetal circulation is unique due to the presence of the placenta, which provides the gas-exchange requirements of the fetus**. The lungs are filled with fluid and, as a result, pulmonary vascular resistance (PVR) is high and the majority right ventricular output bypasses the pulmonary circulation through the ductus arteriosus (DA). Therefore, the placenta supplies both left and right ventricular output, with left ventricular output being support by blood flow through the foramen ovale (FO). Middle Panel: With clamping of the umbilical cord you immediately remove placental supply of right and left ventricular output, resulting in an instantaneous reduction to combined ventricular output (CVO) by ~50%. Since the lungs are still filled with fluid, PVR is high thus right ventricular output is still diverted away from the pulmonary circulation, through the DA. Right Panel: Removal of lung liquid and aeration of the lung results in a rapid fall in PVR and a subsequent increase in PBF, allowing 100% of right ventricular output to enter the lungs. This allows the pulmonary circulation to replace the placenta as the source of left ventricular output. There is also a reversal in pressure gradient and thus direction of blood flow across the ductus arteriosus (from left-to-right to right-to-left), allowing for up to 50% of left ventricular output to enter the pulmonary circulation, thus further stabilizing the upper body circulation.

The fetal pulmonary and systemic circulations are not separate as they are in adults and the placenta is connected in parallel with the fetal lower body, thereby forming part of the fetal systemic circulation (30). Thus, a large proportion of venous return to the fetal heart is derived from the placenta, which either passes through the ductus venous (DV) or liver before entering the inferior vena cava (IVC) (30, 31). Umbilical venous blood passing through the DV undergoes little mixing in the IVC and the majority passes through the foramen ovale (FO) directly into the left atrium (Figure 1). As a result, blood exciting the left ventricle has higher oxygen saturation levels than blood exciting the right ventricle, which gives rise to the higher oxygen saturation levels in pre-ductal arteries compared to post-ductal arteries (31). In the fetus, the majority (~90%) of blood exiting the right ventricle bypasses the lungs, because of the high PVR, and passes through the ductus arteriosus (DA) into the descending aorta (30, 32). Blood flow in this direction is referred to as right-to-left (R-to-L) shunting, whereas blood flow from the systemic into the pulmonary circulation is referred to as left-to-right (L-to-R) shunting (Figure 1). Blood flow into the fetal lungs only occurs briefly during systole whereas throughout diastole blood flows in a retrograde manner away from the lungs (32). This retrograde PBF exits the pulmonary circulation through the DA, which accounts for the high R-to-L DA flow during diastole (32).

During fetal life, the left ventricle receives much of its preload from the umbilical circulation via the DV and FO, because PBF is usually low during fetal life (Figure 1). However, fetal PBF is not always low and can markedly increase (up to fourfold) during periods of accentuated fetal breathing movements (33). As FO flow and PBF are inversely related (34), it is likely that these two sources of preload counter-balance each other to ensure a continuous supply of blood for the left ventricle. Nonetheless, PBF usually provides only a small fraction of venous return and preload for the left ventricle, with the majority coming from umbilical venous return via the DV and FO (30, 31). As such, the loss of umbilical blood flow due to ICC markedly reduces venous return and preload for both ventricles at birth if PBF does not simultaneously increase (6, 32). In lambs, RVO decreases by 50% with ICC and, due to the loss of the low resistance placental circulation, systemic vascular resistance markedly increases resulting in a rapid (within four heart beats) increase in arterial pressure (6, 32). The resulting increase in afterload combined with the reduced preload results in a reduction in cardiac output (6, 32).

Recently, published normograms demonstrate that heart rates in normal healthy infants are reduced immediately after birth and rapidly increase within a few minutes (35). As low heart rates at this time are usually attributed to an asphyxia-induced vagally mediated bradycardia (36), the common assumption is that infants with a low heart rate are asphyxic. However, as these infants were not considered to be hypoxic at birth, other physiological factors may have contributed to the low heart rates observed (35). Indeed, a reduction in preload combined with an increase in afterload caused by ICC, are possible contributing factors.

#### The cardiovascular consequences of lung aeration

After birth, aeration of the lung causes a marked decrease in PVR and increase in PBF (20). Although the precise mechanisms involved are still unclear, the cardiovascular changes resulting from this increase in PBF are well established (6, 32). Due to a substantial decrease in PVR, the distribution of RVO rapidly changes so that the pulmonary circulation becomes its primary recipient (6, 32). Combined with an increase in systemic vascular resistance caused by cord clamping, the decrease in PVR reverses the pressure gradient across the DA, causing flow to reverse and become predominantly L-to-R in both sheep (32) and human beings (37, 38) (Figure 1). As a result, left ventricular output becomes a significant source of PBF immediately after birth with up to 50% being derived from the left ventricle (32). Although net flow across the DA is L-to-R, instantaneous flow is mostly bidirectional with R-to-L flow occurring briefly during early systole (32, 37) (Figure 1). Throughout late systole and diastole, flow through the DA is mostly L-to-R, which results in high diastolic flows in the pulmonary arteries. These characteristic flow patterns likely arise due to the anatomical relationship of the DA to both the pulmonary artery and aorta. That is, during systole the pressure wave emanating from the right ventricle likely reaches the pulmonary artery/DA junction before the pressure wave exiting the left ventricle reaches the aorta/DA junction. This results in an initial pressure gradient across the DA that favors R-to-L flow until the pressure wave from the left ventricle reaches the aortic end of the DA. At this time the pressure gradient reverses, causing L-to-R flow through the DA, with the resulting turbulence possibly contributing to closure of the DA after birth.

Despite a marked and persisting decrease in right ventricular output caused by UCC, PBF markedly increases immediately after birth. This is due to both the redirection of right ventricular output into the lungs and the large contribution (~50%) of flow from L-to-R shunting across the DA (32). This highlights the importance of L-to-R shunting through the DA to PBF and therefore left ventricular preload immediately after birth, effectively allowing the two ventricles to continue to work at different output levels, while the DA remains open (Figure 1). Although a persisting DA is problematic for infants, it can be argued that it is desirable for the DA (and FO) to briefly (~2–3 h) remain patent immediately after birth. This would allow the outputs of the two ventricles to stabilize and gradually equalize before the DA closes, which would enable a smoother transition to an “in-line” inter-dependent circuit.

#### The cardiovascular benefits of lung aeration before umbilical cord clamping

As the supply of preload for the LV must change from umbilical venous return to pulmonary venous return after birth, establishing ventilation in an infant before its cord is clamped allows PBF to increase before umbilical venous return is lost (6). As a result, the supply of preload for the left ventricle can immediately switch from umbilical venous return to pulmonary venous return without an intervening period of reduced venous return and low cardiac output (Figure 1). An experimental study has recently confirmed these concepts and has detailed the cardiovascular benefits associated with UCC after ventilation onset (6). For instance, following lung aeration and the decrease in PVR, flow through the pulmonary circulation can become an immediate alternative pathway for output from both right and left ventricles (via L-to-R flow through the DA). As a result, the increase in systemic arterial pressure associated with UCC is greatly diminished as is the reduction in both right and left ventricular output. Instead, all cardiovascular parameters examined underwent a comparatively very stable transition, thereby avoiding the large swings in cardiac output, arterial pressure, and cerebral blood flow (6). With regard to the latter finding, if cord clamping precedes ventilation onset, the rapid (within four heart beats) increase in arterial pressure rapidly increases cerebral blood flow, because over this time frame cerebral blood flow is pressure passive. Flow then rapidly decreases again due to a decrease in cardiac output before increasing again after ventilation commences due to an increase in PBF and cardiac output. These unprotected large swings in pressure and flow likely increase the risk of IVH, particularly in preterm infants that have an immature cerebral vascular autoregulatory capacity (39). As clamping the umbilical cord after ventilation onset greatly minimizes these changes (6), this mechanism potentially explains why DCC results in a reduction in the incidence of IVH.

These observations underpin the well established and widely taught concept that establishing effective ventilation at birth is the cornerstone of neonatal resuscitation (40). That is, establishing effective ventilation not only allows pulmonary gas exchange, but is also vital for maintaining cardiac output by increasing PBF so that it can supply the necessary preload for the left ventricle. Most infants, including preterm infants, do this themselves by crying and commencing breathing as soon as they are delivered, before the caregiver has time to clamp the cord (41). However, in infants that are apneic at birth, the perceived “urgent” need to establish ventilation can lead to rapid UCC so that the infant can be removed to a separate location for resuscitation (10). However, doing this will necessarily lead to a reduction in cardiac output for the indefinite period of time that it takes to initiate pulmonary ventilation and to stimulate an increase in PBF.

## Third Stage of Labor

The maternal third stage of labor is the period of time between delivery of the infant and delivery of the placenta and can be managed in two different ways; either expectantly or actively. The active approach is currently the most commonly employed method and involves administration of an uterotonic agent upon delivery of the infant’s anterior shoulder. This is followed by immediate UCC upon the infant’s delivery and controlled cord traction to separate and deliver the placenta. The aim is to shorten the duration of the third stage, which is a strategy that is thought to be associated with lower rates of PPH. As such, active management is highly encouraged (42, 43). In contrast, expectant management is not time-critical and may involve delayed or no uterotonic administration and cord clamping is delayed until after the cord ceases pulsing or signs of placental separation are evident. Spontaneous delivery of the placenta is encouraged, resulting in an overall prolongation of the third stage of labor (43).

Delaying UCC during the third stage of labor is thought to result in “placental transfusion,” allowing a time-dependent net transfer of blood volume from the placenta into the infant (5, 44). It has been proposed that the rate of transfusion follows an exponential decay curve, with 25% of it being complete by 15 s and 50% by 60 s with flow ceasing in most infants by 2–3 min, although it can extend for up to 5 min in some (45–48). However, if a net transfer of blood from placenta to the infant does occur, it is unlikely to simply be time dependent as physiologists have been studying exteriorized fetuses for over 60 years (36). In such instances, blood flow in the umbilical cord can continue for hours and maintain physiological homeostasis throughout that time. As such the net transfer of blood and the cessation of flow in the cord are likely to be determined by various physiological factors rather than the delivered time. Potential physiological factors include uterine contractions following uterotonic administration, vertical positioning of the neonate either above or below the placenta, and the onset of pulmonary ventilation. Unfortunately, other than pulmonary ventilation, the potential confounding influences of these factors have not been closely examined. Nevertheless, expectant management and DCC are associated with higher birth weights most likely due to greater neonatal blood volumes (43); volumes can increase by ~30% according to some studies whereupon DCC is extended (46, 48).

## Key Determinants of Effective Placental Transfusion with DCC

Although current evidence favors the practice of DCC, particularly in preterm infants (44, 49), the evidence is not definitive and, as such, current recommendations on the timing of cord clamping for preterm infants are not explicit (11). While this can be partly explained by inconsistencies in the experimental protocols employed, an overall lack of scientific information to guide the clinical studies has been a major limitation.

The reported (5, 44, 49–54) benefits of DCC include

(1) an increase in systemic blood volume and hematocrit in the immediate newborn period;
(2) a reduced risk of anemia and need for blood transfusions;
(3) improved iron stores and lower rates of iron deficiency, lasting for 3–6 months after birth;
(4) higher birth weights, most likely due to higher neonatal blood volumes;
(5) lower risks of IVH (all grades) and necrotizing enterocolitis.

Potential risks associated with DCC (5, 50, 55) include

(1) higher rates of hyperbilirubinemia (jaundice) in both term and preterm infants although this did not lead to a significantly increased need for more phototherapy;
(2) a higher incidence of polycythemia (venous hematocrit > 65%), but no infants were found to develop symptoms or complications related to the condition (56).

While these clinical studies have highlighted an increase in blood volume as the major benefit of DCC, the actual benefits could potentially be far more extensive. To some extent, this is indicated by the lower risk of IVH, which is difficult to explain simply in terms of blood volume. Indeed, the insights provided by recent experimental studies provide a new understanding of the potential benefits of DCC as well as a different approach for determining the appropriate timing of DCC after birth (6). That is, instead of delaying cord clamping for a set period of time, these studies indicate that the timing of cord clamping should be based on the infant’s physiology rather than an arbitrary period of time. The likelihood of previous clinical studies uncovering all benefits of DCC, when cord clamping is based on a set period of time, is low as the physiological changes that underpin the potential benefits occur at different times in different infants. Indeed, one of the commonest reasons for why umbilical cords are hastily clamped at birth is to initiate respiratory support. However, it could be argued that these infants would receive the greatest benefit if the respiratory support was provided while the umbilical cord remained attached to the placenta (6).

In addition to the onset of pulmonary ventilation and the associated increase in PBF, there are a number of other physiological factors that could impact on the benefits of DCC. These include uterine contractions and the vertical position of the infant with respect to the placenta.

### Uterine contractions

Intramuscular uterotonic injection (either syntocinon or syntometrine) upon delivery of the infant’s anterior shoulder is the recommended practice in Australia (43). Numerous clinical trials, summarized in a Cochrane review, have shown that uterotonics successfully lower the risk of PPH following placental separation by preventing uterine atony, the commonest cause of PPH (42, 43). While the placenta is attached it has been suggested that uterine contractions “squeeze” blood toward the fetus enabling placental transfusion (45). However, this suggestion is not consistent with the interpretation of normal fetal heart rate decelerations and recovery observed during labor (57). Indeed, fetal heart rate deccelerations, which are common in second stage labor, are thought to be due to uterine contractions causing a reduction in uterine and umbilical blood flows (57). Further, the mild rebound fetal tachycardia following a contraction is thought to result from differential compression of umbilical veins and arteries. This results in earlier occlusion and later release of the more compliant venous vessels, compared to arteries, tending to cause blood accumulation in the placenta (57). The release of this blood back into the fetal circulation at the end of the contraction is thought to increase venous return and drive the mild rebound tachycardia that is commonly observed and termed a “shouldering affect”. This understanding is consistent with the finding that oxytocin administration before UCC significantly reduces infant birth weight due to a reduction in neonatal blood volume (43).

Although uterotonic administration reduces the risk of PPH (43), the timing of administration during third stage is not a significant factor. Indeed, oxytocin administration before or after placental delivery does not influence the incidence of PPH, retained placenta, duration of third stage of labor, post-partum blood loss or incidence of maternal hypotension (42). However, the potential for uterotonic administration to influence umbilical vascular resistance and flows will likely have a profound impact on the cardiovascular benefits associated with DCC. Although there is currently no experimental evidence confirming this contention, it is possible that future recommendations will include uterotonic administration only after UCC.

### Vertical position of infant relative to the placenta; effect of gravity

As umbilical venous pressures are low, it is commonly assumed that gravity and resistance will influence umbilical blood flow between the infant and its placenta. Thus, it is assumed that placing the infant above the level of the placenta will facilitate flow into the placenta whereas placing the infant below the placenta will facilitate flow into the infant. Previous studies (47, 48, 58) indicate that placental transfusion is maximized when neonates are placed below the level of the placenta (at least 10 cm below the introitus) (58). While the effects were explained by the ability of gravity to assist or reduce the placental transfusion caused by uterine contractions, the hemodynamics are likely to be considerably more complex. Although a recent clinical trial has found no adverse effects for placing the infant above the placenta (59), no scientific studies detailing the effect of gravity on umbilical blood flows (arterial and venous) have been conducted to inform our understanding of what the likely effects of gravity are. Current common practice is to place the infant above the level of the placenta on the mother’s abdomen or chest after birth and while this does not appear to influence infant outcome (59), the science underpinning this finding is unknown.

### Umbilical cord milking

Milking or stripping the umbilical cord following clamping is reported to have beneficial effects for the newborn by assisting or augmenting the placental transfusion, thereby reducing the time required for DCC (60–64). Indeed, studies have shown that multiple cord milkings increase hemoglobin and serum ferritin levels at 6 weeks after birth in term and near-term infants and led to significantly higher blood pressures (although within a normal range) over the initial 48 h after birth (63, 64). However, there is the potential for this procedure to cause harm to the infant and studies are urgently required to critically examine the consequences. For example, cord stripping could potentially cause the rapid release of a large bolus of blood into the infant’s circulation. Alternatively, multiple strippings of the cord could possibly release cytokines and/or cellular debris into the infant’s circulation.

## Critical Gaps in the Literature

Although a considerable amount of data have been published on DCC, the absence of rigorous scientific data has made it difficult to reach a consensus upon the precise benefits and appropriate timing of DCC and the factors that influence this. Recent experimental evidence indicates that DCC until after the lung has aerated and PBF has increased greatly increases the stability of the cardiovascular transition at birth (6). In particular, it greatly mitigates the large swings in cardiac output and arterial pressure and flow caused by UCC, thereby potentially reducing the risk of IVH. However, we need to know how numerous other factors, such as gravity influences the placental to infant transfusion of blood and the cardiovascular transition at birth. Furthermore, we need to know how uterine contractions influence the benefits of DCC to ascertain the most appropriate timing of uterotonic administration. This may require revisiting how the third stage of labor is managed, with the focus on care practices that benefit both the mother and infant.

## Conclusion

Delayed umbilical cord clamping is a subject that has attracted much debate and is clearly a topic that could greatly benefit by the application of further scientific experimentation. Although current guidelines recommend a fixed time interval between birth and UCC, this is unlikely to optimize the benefits for individual infants. Instead, the decision for clamping the umbilical cord after birth should probably be based on the infants’ physiology rather than an arbitrary period of time. This suggestion is based on the markedly improved cardiovascular stability associated with cord clamping after ventilation onset and the increase in PBF. The reasons and logic behind this suggestion has been well documented and described, but further experimentation is required to uncover additional variables that may impact on the benefits of DCC. In particular, we need to understand how gravity and utertonic administration impacts on the infants cardiovascular transition if cord clamping is delayed, which could potentially lead to revisiting the clinical practices for managing the third stage of labor.

## Conflict of Interest Statement

The authors declare that the research was conducted in the absence of any commercial or financial relationships that could be construed as a potential conflict of interest.
